# MiRNA polymorphisms affect the prognosis of gastric cancer: insights from Xianyou, Fujian

**DOI:** 10.3389/fonc.2024.1355270

**Published:** 2024-05-15

**Authors:** Ping Wu, Yuling Zhang, Yanping Lyu, Jingwen Chen, Yu Jiang, Jianjun Xiang, Baoying Liu, Chuancheng Wu

**Affiliations:** ^1^ Department of Preventive Medicine, School of Public Health, Fujian Medical University, Fuzhou, China; ^2^ Department of Pulmonary and Critical Care Medicine, Fujian Medical University Union Hospital, Fuzhou, Fujian, China

**Keywords:** miRNA, single nucleotide polymorphism, prognosis, gastric cancer, rs17502941

## Abstract

**Introduction:**

Gastric cancer, characterized by high incidence and substantial disease burden, has drawn continuous attention regarding its occurrence and prognosis. Genetics plays a crucial role in influencing the prognosis of gastric cancer, and single nucleotide polymorphisms are closely associated with the occurrence, development, and prognosis of this malignant tumor. Our study aims to conduct survival analysis on patients carrying different single nucleotide polymorphisms, exploring the relationship between miRNA single nucleotide polymorphisms and the prognosis of gastric cancer.

**Methods:**

Genetic data from 344 patients in Xianyou, Fujian, formed the basis of our study. We delineated the survival rate and median survival time, utilizing the log-rank test and COX regression analysis as statistical tools.

**Results:**

Upon stratifying the data by sex or operation, it was discerned that the GG genotype at MSH2 rs17502941 independently posed a heightened risk for gastric cancer. Other stratification analyses suggested that the subsequent single nucleotide polymorphisms were correlated with patient prognosis: rs17502941, rs884225, rs1468063, rs7143252, and rs2271738.

**Discussion:**

The outcomes of this study strongly suggest that miRNA polymorphisms significantly influence the survival time of gastric cancer patients and can serve as effective predictors for the prognosis of gastric cancer.

## 1 Introduction

According to cancer incidence and mortality data for the year 2018 from the International Agency for Research on Cancer (IARC), gastric cancer constituted 8.2% of all cancer-related deaths (1). A substantial number of patients receive a diagnosis of advanced-stage cancer, indicative of a low rate of early detection (2). Simultaneously, the prognosis of gastric cancer is intricately linked to various factors, encompassing the degree of differentiation, sex, tumor microenvironment, and the epithelial-mesenchymal transition (EMT) signature associated with metastasis (3–5). Heredity stands out as a significant factor influencing the prognosis of gastric cancer, with single nucleotide polymorphism closely intertwined with the onset, progression, and prognosis of this malignancy. A comprehensive meta-analysis indicates that diminished microRNA expression is detrimental to patient prognosis and may expedite the progression of gastric cancer (6). Discrepancies in miRNA blood levels have been identified in patients with gastric cancer (GC), affirming the utility of miRNA expression as a diagnostic and prognostic biomarker for this condition (7). Moreover, investigations have proposed a close association between miRNA and multidrug resistance (8, 9). There is suggestive evidence that miR-214 can regulate the expression of FGF9, impeding the migration and invasion of gastric cancer (GC) cells. Conversely, miR-519a-3p has been implicated in promoting liver metastasis in gastric cancer and is associated with an unfavorable prognosis (10, 11). Several studies have identified miRNA-21 rs1292037 and miR-149 rs2292832C/T as potential prognostic indicators for hepatocellular carcinoma (12, 13). Additionally, investigations have linked miR-34 rs4938723 and miR-195-5p to the prognosis of colorectal cancer (14, 15). Moreover, research findings indicate that single nucleotide polymorphisms in miRNAs are correlated with the prognosis of various types of tumors (16–19). While numerous studies have established associations between miRNA single nucleotide polymorphisms and the prognosis of various types of tumors, limited attention has been directed toward exploring the connection between miRNA single nucleotide polymorphisms and the prognosis of gastric cancer. Our research complements the existing knowledge body in this specific domain.

Our study aimed to scrutinize the correlation between miRNA polymorphisms and the prognosis of gastric cancer. We meticulously selected 11 miRNA loci and assessed their impact on gastric cancer prognosis using four distinct models: Co-dominance, Allele gene, dominant model, and recessive model. Subsequently, a stratified analysis was conducted based on parameters such as sex, age, location, TNM stage, the performance of surgery, and administration of chemotherapy. Multivariate COX analysis was then applied to sites demonstrating statistical significance in the stratified analysis, thereby delving into the factors exerting independent risk effects on the prognosis of gastric cancer.

## 2 Materials and methods

### 2.1 Study populations

This research encompassed the distribution of 555 questionnaires, of which 96 cases posed challenges for investigation due to reasons such as removal, denial of dis-ease, and rejection of examination. (recovery rate: 82.7%) Following a thorough examination of the questionnaires, 115 incomplete and unqualified counts were excluded, and ultimately, 344 questionnaires were finally adopted. Therefore, our study included 344 patients, with peripheral blood samples collected from each patient. The inclusion criteria were as follows: (1) voluntary participation in the study and signing of informed consent by the researchers, (2) patients confirmed by endoscopic biopsy or surgical specimens to have gastric adenocarcinoma, (3) residing in Xianyou for more than 10 years, (4) confirmed between April 2013 and November 2017, with the final follow-up conducted in December 2022. Exclusion criteria were: (1) patients simultaneously diagnosed with other types of tumors besides gastric adenocarcinoma, (2) severe mental illness or poor compliance leading to inability to follow-up, (3) severe genetic diseases, including chromosomal number and structural abnormalities. The histological type of tumors was evaluated according to the standards of the World Health Organization (WHO), and tumor staging was classified according to the guidelines outlined in the American Joint Committee on Cancer Staging Manual (7th edition).

### 2.2 Selection of polymorphism loci

**Figure 1:**
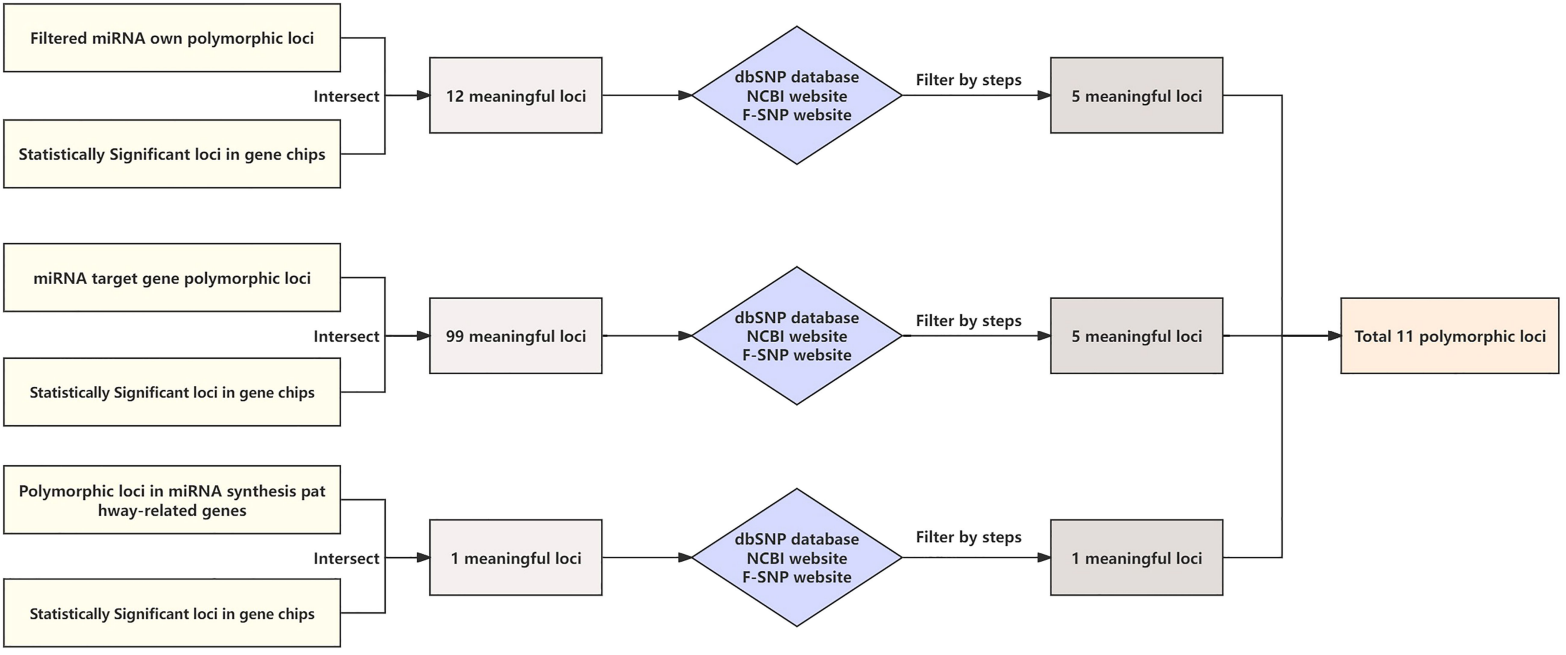
Flowchart for selecting required loci.

Previous studies have identified 112 loci associated with gastric cancer, including 12 miRNA polymorphic loci, 99 miRNA target gene polymorphic loci, and 1 miRNA synthesis pathway gene polymorphic locus (**Figure 1**). To further identify the miRNA-SNPs most closely related to gastric cancer, we selected 11 loci for this study through the following steps:

1. Initially, utilizing the dbSNP database (http://www.ncbi.nlm.nih.gov/projects/SNP/), we determined the minimum allele frequency (MAF) of each SNP locus in the Chinese population. Loci with MAF between 0.15 and 0.40 were chosen.
2. Subsequently, based on information from the NCBI website (https://pubmed.ncbi.nlm.nih.gov/), we selected loci associated with gastrointestinal (GI) tumors.
3. Finally, using the F-SNP website (http://compbio.cs.queensu.ca/F-SNP/), we screened for loci with corresponding functions.

### 2.3 SNP genotyping

The 11 selected miRNA candidate loci were examined using the Sequenom Mass ARRAY SNP method. The process involved the following steps:

1. Designing Primers:

Design PCR amplification primers and single-base extension primers for SNP locus to be tested using Genotyping Tools and Mass ARRAY Assay Design software (**Table 1**).

**Table 1:** Primers of loci.

Polymorphisms	Amplification primers	Single-base extension primers
rs7143252	F:ACGTTGGATGATTGGGTGAGTCACAGCTTG R:ACGTTGGATGAGGCATGTGGACAATGCACG	ggaatTGTGAGCTAGTCTAGTCTAA
rs10413288	F: ACGTTGGATGTGGTCAAAGAAAATTCCAGG R: ACGTTGGATGCAGAAAGTGCTTCCCTCAAG	aaAGCCTGACATCTTCTTGCTC
rs9536676	F: ACGTTGGATGCAGTTATTGTAGTGTTATGC R: ACGTTGGATGCAGTAATTGCTTACCATTCC	TCCATTTTTCTATCCTTTGAC
rs4713714	F: ACGTTGGATGGGAAAAAGTGCTGATGGTG R: ACGTTGGATGAAGATGCCAGCCTACTAGGG	TGGTGCTGCTTCCTCC
rs2724377	F: ACGTTGGATGGAAGTAGGCAAGATCTGCAC R: ACGTTGGATGGTTCAGTTTATCATGTCTGTG	TCAGGACCCACTTCTTATC
rs884225	F:ACGTTGGATGATGAGCGTTAGACTGACTTG R:ACGTTGGATGGCTGATTTCATGACAGCAAG	acagTGTTTTGAAACTCAGTATGC
rs10277413	F: ACGTTGGATGTCGCTGCCAGATGATTGTTC R:ACGTTGGATGCTAATTACGGAGAAGGCTGG	gggaGTCAGAAACCTGCAGGGAC
rs1468063	F: ACGTTGGATGTCTGGATTTAGGAATTGCTC R: ACGTTGGATGCGTAGGATAGTAGTAAGGAG	TTGCTCTTGTCATACCC
rs17502941	F: ACGTTGGATGACAAACCCACACCTTTCAGC R: ACGTTGGATGGGCTAGCAGAGCAGACACA	GCAGACACACAGCTC
rs11125144	F: ACGTTGGATGTAGGGAATGGCAGGGTTTGG R: ACGTTGGATGTCACCCTAAGCCAACTCTTG	GCTACCACTGCTATGAGAC
rs2271738	F: ACGTTGGATGATCTCTTCTTGCCGATCGGG R: ACGTTGGATGACGGACAATCAGACCTCAAC	gagCGGGCGCCGACCTAGCAGT

2. PCR Amplification:

Perform PCR amplification reactions for the selected loci.

3. Product Processing:

Process the PCR amplification products.

4. Mass ARRAY Dispensing:

Initiate the Mass ARRAY RS1000 dispenser and deposit the processed product onto the SpectroCHIP (Sequenom) chip.

5. SpectroCHIP Detection:

Employ Mass ARRAY MALDI-TOP to detect the SpectroCHIP, capturing the type and output of results.

6. Result Analysis:

Analyze the results using TYPER4.0 software.

This methodology allows for the precise genotyping of the selected miRNA candidate loci.

### 2.4 Quality control

#### 2.4.1 Quality of questionnaires

Throughout the on-site investigation, the inspectors meticulously adhered to the questionnaire requirements. Following the survey, the questionnaire data underwent a thorough examination. Any incomplete questionnaires were promptly supplemented with as much information as possible. To ensure data accuracy, a double data entry method was employed, involving logical corrections and consistency testing. Additionally, a random sample of 10% of the entries underwent further review to enhance the overall quality and reliability of the data.

#### 2.4.2 Quality of genotyping

Dish QC (DQC) serves as a pivotal metric for evaluating the quality of genotyping. This assessment relies on comparing the distribution of signal values with background signal values, where a greater difference signifies a more effective experimental process and higher genotyping quality for the respective sample. In this experiment, all DQC results exceeded 0.82, indicating excellent genotyping quality. Genotypes were determined in a blinded manner, and 2% of the samples underwent random selection for repeated testing. The concordance rate in these repeated tests was found to be 98.47%. This robust quality control process ensures the reliability and accuracy of the genotyping results.

### 2.5 Statistical analysis

Survival rates for different genotypes of the same polymorphic locus in the 1st, 3rd, and 5th years were derived from the mortality table. The Kaplan-Meier method was employed to calculate the survival time of patients with different genotypes, and corresponding survival curves were graphically represented. The log-rank test was then applied to analyze the correlation between single nucleotide polymorphism and the prognosis of gastric cancer. Hazard ratios (HRs) and 95% confidence intervals (CIs) were computed using both univariate and multivariate COX regression modeling methods.

For statistical analysis, the significance level (α) was set at 0.05, and all P values were based on two-sided tests. The statistical software utilized for these analyses was SPSS 24.0, ensuring robust and standardized statistical evaluation of the data.

### 2.6 Ethics approval

The study was conducted in accordance with the Declaration of Helsinki, and approved by the Ethics Committee of Fujian Medical University, China (No. 97,2014).

## 3 Results

### 3.1 Characteristics of study populations

Among the 344 patients included in the study, there were 252 male patients and 92 female patients, ranging in age from 36 to 96 years, with an average age of (69.15 ± 9.34) years and a median age of 69 years. The survival rates for the 344 gastric cancer patients were recorded at 73.47%, 45.71%, and 39.67% in the 1st, 3rd, and 5th years, respectively, with a median survival time of 30.00 months.

To explore the relationship between the overall condition and prognosis of patients with stomach cancer, the aforementioned statistical methods were employed. It was observed that age, TNM stage, and whether surgery was performed were all correlated with the prognosis of gastric cancer patients (P<0.05) (**Table 2**).

**Table 2:** Relationship between basic characteristics and prognosis of patients.

Variables	n	MST (M)	Survival rate (%)			HR (95%CI)	Log-rank P
			1-year	3-year	5-year		
Sex							
Male Female	252 92	29.00 32.00	72.56 75.96	45.39 46.59	38.84 42.00	1 0.842 (0.707,1.327)	0.841
Age							
≦65	120	55.00	84.10	58.86	47.04	1	0.003*
65-	224	24.00	67.79	38.75	35.65	1.580 (1.163, 2.145)	
Location							
Non-cardia	179	28.00	72.07	44.68	39.76	1	0.503
Cardia	165	31.00	75.00	46.84	39.71	0.91 (0.688, 1.203)	
TNM stage							
1	51	–	94.12	81.40	78.59	1	<0.001*
2	166	75.00	91.57	62.45	54.34	**2.580 (1.330, 5.005)**	
3	127	10.00	41.27	9.33	5.52	**12.305 (6.373, 23.796)**	
Operation							
No	76	8.00	35.53	5.24	3.50	1	<0.001*
Yes	268	64.00	84.27	57.17	49.92	**0.190 (0.140, 0.258)**	
Chemotherapy							
No	140	25.00	65.00	41.21	37.24	1	0.707
Yes	204	33.00	79.31	48.81	41.23	0.758 (0.572, 1.003)	
Radiotherapy							
No	245	31.00	72.95	46.11	42.24	1	0.592
Yes	99	28.00	74.75	44.73	30.93	1.086 (0.800, 1.474)	

All bold values and symbol “*” indicates P < 0.05, and the HR is significant.

Mean survival time was utilized in replacement of MST when it could not be computed.

### 3.2 Relationship between polymorphism and prognosis of gastric cancer

The study incorporated a total of 11 miRNA polymorphic sites, with detailed information provided in **Table 3**. The results of the statistical analysis revealed a significant difference in the survival time associated with the A mutant gene of the polymorphic locus miRNA-1297 rs9536676 in comparison to the wild-type gene G. Specifically, the risk of death for patients carrying the A allele was 1.258 times higher than that of patients carrying the G allele (HR=1.258, 95% CI=1.000-1.581, Log-rank P = 0.046). In the case of the polymorphic locus *MSH2* rs17502941, survival times exhibited statistical significance in the recessive model (AA+AG/GG). Patients carrying the GG genotype had a 1.329 times higher risk of death compared to those carrying the AA/AG genotype (HR=1.329, 95% CI=1.006-1.756, Log-rank P=0.043) None of the other polymorphic loci were found to be associated with the prognosis of gastric cancer (**Tables 4**, **5**).

**Table 3:** Basic information of 11candidate miRNA SNP locus.

No.	SNP ID	Gene name	Chromosome	Location	H-W P	MAF
1	rs7143252	miRNA-379	14	101487621	0.331	0.211
2	rs10413288	miRNA-519b	19	54197633	0.823	0.392
3	rs9536676	miRNA-1297	13	54886472	0.122	0.227
4	rs4713714	miRNA-7159	6	33865380	0.721	0.297
5	rs2724377	miRNA-29c	1	207974818	0.373	0.170
6	rs884225	*EGFR*	7	55274048	0.931	0.187
7	rs10277413	*EGFR*	7	55238464	0.481	0.377
8	rs1468063	FAS	10	90775291	0.812	0.243
9	rs17502941	*MSH2*	2	47762396	0.101	0.389
10	rs11125144	*MSH2*	2	47562636	0.830	0.159
11	rs2271738	*AGO2*	8	141566311	0.671	0.341

**Table 4:** The relationship between miRNA-1297 rs9536676 and prognosis.

rs9536676	n	MST (M)	Survival rate (%)			HR (95%CI)	Log-rank P
			1-year	3-year	5-year		
Codominance							
GG	217	36.00	75.00	50.13	36.09	1	0.161
AG	105	20.00	67.62	40.88	32.29	1.287 (0.950-1.743)	
AA	22	21.00	86.36	27.27	27.27	1.398 (0.829-2.359)	
Allele gene							
G	539	34.00	73.56	48.32	35.35	1	0.046*
A	149	21.00	73.15	36.34	29.84	**1.258 (1.000-1.581)**	
Dominant model							
GG	217	36.00	75.00	50.13	36.09	1	0.060
AG+AA	127	21.00	70.87	38.15	30.78	1.308 (0.985-1.736)	
Recessive model							
GG+AG	322	31.00	72.59	47.11	34.85	1	0.327
AA	22	21.00	86.36	27.27	27.27	1.287 (0.772-2.148)	

All bold values and symbol “*” indicates P < 0.05, and the HR is significant.

**Table 5:** The relationship between *MSH2* rs17502941 and prognosis.

rs17502941	n	MST (M)	Survival rate (%)			HR (95%CI)	Log-rank P
			1-year	3-year	5-year		
Codominance							
AA	31	29.00	87.10	43.83	38.96	1	0.127
AG	146	36.00	76.63	52.30	37.25	0.980 (0.579-1.656)	
GG	167	23.00	68.17	40.16	30.74	1.307 (0.782-2.182)	
Allele gene							
A	208	36.00	79.76	50.02	37.59	1	0.077
G	480	27.00	70.74	43.85	32.59	1.213 (0.976-1.508)	
Dominant model							
AA	31	29.00	87.10	43.83	38.96	1	0.593
AG+GG	313	30.00	72.12	45.82	33.64	1.143 (0.695-1.880)	
Recessive model							
AA+AG	177	36.00	78.47	50.99	37.44	1	0.043*
GG	167	23.00	68.17	40.16	30.74	**1.329 (1.006-1.756)**	

All bold values and symbol “*” indicates P < 0.05, and the HR is significant.

### 3.3 Stratified analysis

In the dominant model, the AG and AA genotypes at miRNA-1297 rs9536676 emerge as risk factors for patients aged over 65 years, male patients, and those diagnosed with cardiac cancer. Similarly, the AG and GG genotypes at miRNA-519b rs10413288 serve as protective factors for patients with cardiac cancer and those who have undergone chemotherapy, as per the dominant model. Notably, the AG and GG genotypes at *MSH2* rs11125144 act as risk factors for female patients (co-dominant and dominant models) but as a protective factor for patients who did not undergo surgery (recessive model).

In the stratified analysis, rs17502941, rs884225, rs1468063, rs7143252, and rs2271738 were all identified as significant factors linked to the prognosis of patients (**Tables 6**–**11**).

**Table 6:** Univariate survival analysis stratified by sex (Male, Female).

Gene locus	Model		n	MST (M)	HR (95%CI)	Log-rank P
rs9536676	Dominant model	GG	160	29.0	1	0.049*
		AG+AA	93	19.0	1.382 (0.995-1.918)	
rs17502941	Recessive model	AA+AG	123	30.0	1	0.034*
		GG	130	21.5	1.415 (1.022-1.960)	
rs11125144	Codominance	AA	48	34.0	1	0.027*
		AG	36	18.5	2.118 (1.195-3.755)	
		GG	7	34.0	1.184 (0.353-3.975)	
	Dominant model	AA	48	34.0	1	0.015*
		AG+GG	43	19.0	1.962 (1.121-3.432)	

*P<0.05.

**Table 7:** Univariate survival analysis stratify by age (>65).

Gene locus	Model		n	MST (M)	HR (95%CI)	Log-rank P
rs9536676	Dominant model	GG	140	27.0	1	0.044*
		AG+AA	84	17.0	1.380(0.987-1.930)	

Symbol “*” indicates P < 0.05, and the HR is significant.

**Table 8:** Univariate survival analysis stratify by region (Non-cardia cancer, Cardiac cancer).

Gene locus	Model		n	MST (M)	HR (95%CI)	Log-rank P
rs884225	Codominance	TT	38	25	1	0.015*
		CT	94	30.5	0.657 (0.405-1.066)	
		CC	47	14.0	1.216 (0.722-2.046)	
	Recessive model	TT+CT	132	27.0	1	0.017*
		CC	47	14.0	1.633 (1.082-2.465)	
rs10413288	Codominance	AA	23	19.0	1	0.043*
		AG	65	30.0	0.602 (0.338-1.074)	
		GG	77	32.0	0.492 (0.278-0.870)	
rs9536676	Dominant model	GG	116	32.0	1	0.045*
		AG+AA	49	20.0	1.520 (0.988-2.337)	
rs1468063	Recessive model	CC+CT	131	30.0	1	0.025*
		TT	34	18.0	1.574 (0.981-2.523)	

Symbol “*” indicates P < 0.05, and the HR is significant.

**Table 9:** Univariate survival analysis stratify by TNM stage (1,2).

Gene locus	Model		n	MST (M)	HR (95%CI)	Log-rank P
rs7143252	Codominance	CC	11	52.0	1	0.040*
		CG	94	35.0	1.938 (0.601-6.246)	
		GG	112	37.5	1.123 (0.345-3.660)	
	Recessive model	CC+CG	105	35.0	1	0.028*
		GG	112	37.5	0.614 (0.395-0.955)	

Symbol “*” indicates P < 0.05, and the HR is significant.

**Table 10:** Univariate survival analysis stratify by operation (Non-operation, Operation).

Gene locus	Model		n	MST (M)	HR (95%CI)	Log-rank P
rs11125144	Dominant model	AA	32	6.5	1	0.039*
		AG+GG	44	9.0	0.616(0.381-0.998)	
rs17502941	Recessive model	AA+AG	138	36.0	1	0.039*
		GG	130	29.5	1.438(1.014-2.039)	

Symbol “*” indicates P < 0.05, and the HR is significant.

**Table 11:** Univariate survival analysis stratify by chemotherapy (Non-Chemotherapy, Chemotherapy).

Gene locus	Model		n	MST (M)	HR (95%CI)	Log-rank P
rs9536676	Codominance	GG	80	31.5	1	0.040*
		AG	50	16	1.444 (0.919-2.269)	
		AA	10	18	2.233 (1.111-4.491)	
	Dominant model	GG	80	31.5	1	0.033*
		AG+AA	60	17.0	1.566 (1.026-2.392)	
rs10413288	Dominant model	AA	30	19.0	1	0.020*
		AG+GG	174	30.0	0.575 (0.357-0.928)	
rs2271738	Recessive model	CC+CT	98	24.5	1	0.014*
		TT	106	30.0	0.631 (0.433-0.918)	

### 3.4 COX multifactor analysis

**Figure 2:**
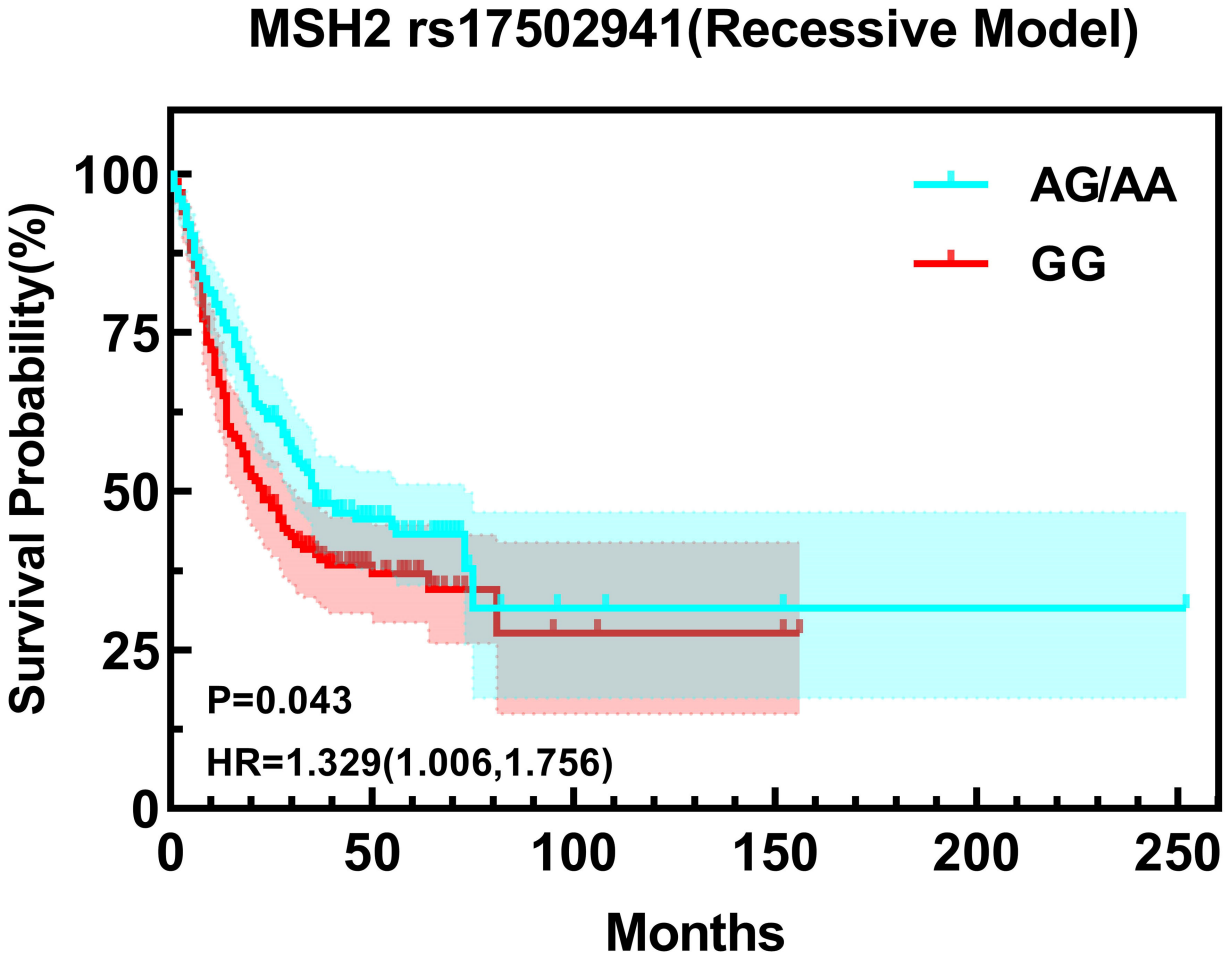
Kaplan-Meier Curve for rs17502941 based on univariate analysis.

**Figure 3:**
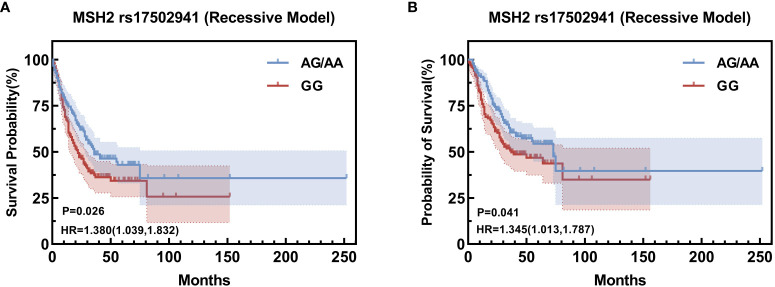
Kaplan-Meier Curve for rs17502941 based on comprehensive COX multifactor analysi, stratified by male **(A)**, stratified by operation **(B)**.

Building upon the insights garnered from the Univariate survival analysis, a comprehensive COX multifactor analysis was conducted specifically for MSH2 rs17502941 (**Figures 2**, **3**). Stratifying the analysis by sex or operation, it was found that the GG genotype at *MSH2* rs17502941 (recessive model) serves as an independent risk factor for the prognosis of gastric cancer (**Tables 12**, **13**).

**Table 12:** COX multifactor analysis stratify by sex (male).

Variables	β	SE	HR (95%CI)	P
TNM stage (1,2 vs 3)	1.534	0.176	4.636 (3.284-6.544)	<0.001
Operation (No vs Yes)	-0.870	0.182	0.419 (0.293-0.599)	<0.001
Radiotherapy (No vs Yes)	0.397	0.163	1.488 (1.082-2.046)	0.014
rs17502941 (GG vs. AA/AG)	0.332	0.145	1.380 (1.039-1.832)	0.026

**Table 13:** COX multifactor analysis stratify by operation (operation).

Variables	β	SE	HR (95%CI)	P
TNM stage (1,2 vs 3)	1.572	0.176	4.815 (3.411-6.798)	<0.001
Radiotherapy (No vs Yes)	0.365	0.162	1.441 (1.050-1.978)	0.024
rs17502941 (GG vs. AA/AG)	0.296	0.145	1.345 (1.013-1.787)	0.041

## 4 Discussion

Our study uncovered a robust association between the single nucleotide poly-morphism of *MSH2* rs17502941 and the prognosis of gastric cancer. Employing COX stratification analysis, we identified that the GG locus of rs17502941 was indicative of a poor prognosis in male patients and those who underwent surgery in a recessive model. Numerous studies have consistently highlighted the intricate relationship between diverse miRNAs and the prognosis of gastric cancer. Elevated expression levels of miR-203, miR-218, and miR-194 have been correlated with a favorable prognosis for gastric cancer, while diminished expression of miR-17-5p and miR-34a serves as a marker for an unfavorable prognosis (6, 20, 21). Furthermore, a diminished expression of PARP-1, a DNA damage response (DDR)-associated protein, has been identified as a prognostic indicator for patients with stage 2 and 3 gastric cancer (22). Given its role as a DDR-associated gene, the low expression of *MSH2* may similarly contribute to an unfavorable prognosis. Numerous investigations have proposed associations between *MSH2* and various cancers, including prostate, colorectal, and ovarian cancers (23, 24), in addition to its linkage with the risk of gastric cancer (25, 26). Additionally, a separate study identified a correlation between the TC+CC genotype of *MSH2* rs2303428 and a diminished survival rate in non-cardia cancers (27). In our investigation, we observed that the GG genotype of *MSH2* rs2303428 stood out as an independent risk factor for an unfavorable prognosis among male, surgical patients, aligning with previous findings. However, in multifactorial analyses, we did not discern their significant effects on the prognosis of gastric cancer.

Earlier studies had implicated these miRNAs in the prognosis of gastric cancer. A research endeavor from Hubei, China, underscored that the diminished expression of miRNA-379 served as an independent risk factor for patients’ prognosis (28). Likewise, a study conducted in Xi’an, China, revealed that miRNA-1297 exhibited low expression in gastric cancer tissues, and this reduced expression independently correlated with compromised disease-free survival for patients (29). However, our investigation sorely yielded statistically significant associations between miRNA-379 rs7143252, miRNA-1297 rs9536676, and gastric cancer prognosis. This discrepancy may be indicative of regional variations in gastric cancer genotyping, and the presence of a complex interplay of confounding factors and genes cannot be discounted. Further research is imperative, as there remains a possibility that multiple loci identified in the univariate analysis are indeed linked to the prognosis of gastric cancer.

Research endeavors have identified risk factors affecting the prognosis of gastric cancer as well as specific loci that could be associated with its prognosis. These findings offer crucial insights, laying the groundwork for further exploration. Some miRNAs related to gastric cancer risk and prognosis have already been collected and arranged by academics, including miR-499, miR-146a, and so on (30), it’s noteworthy that the loci unveiled in our study are not among them. This discrepancy suggests, on the one hand, the significance of our research, and on the other hand, underscores the vast untapped potential within this field. Global epidemiological studies on gastric cancer have unveiled intriguing patterns, emphasizing the distinct genetic landscape in Asian populations. Notably, Asians exhibit a heightened frequency of IL-10 and IL-17 gene diversity. This diversity, in con-junction with environmental factors and lifestyle habits, collectively influences the risk and prognosis of gastric cancer (31). Moreover, specific regional variations have been observed, such as the unique characteristics of gastric cancer in the Tibetan Plateau compared to other regions. This dissimilarity encompasses differences in incidence rates, mutation types, and molecular variations among patients from the highlands and Western countries, as well as Han Chinese and ethnic minorities, have different molecular variations in their mutated gene, For instance, an analysis revealed a notable discrepancy in notch2 mutations, with only one out of 12 Han Chinese exhibiting this mutation compared to seven out of 18 ethnic minorities (32). These findings underscore the imperative need for region-specific sequencing efforts, particularly in areas marked by elevated gastric cancer rates.

Gaining insight into diverse mutation types and single nucleotide polymorphisms across different regions not only establishes a theoretical framework but also lays the molecular foundation for the development of miRNA therapy in gastric cancer. This breakthrough holds the promise of tailoring treatments according to the distinct mutation profiles of patients in various regions. Furthermore, it paves the way for the creation of genetic databases encompassing patients from diverse geographical areas. These databases will serve as valuable reservoirs of background information, facilitating future investigations into the underlying molecular mechanisms. We anticipate that on-going and advanced research endeavors will propel miRNA therapy for gastric cancer from theory to reality. This therapeutic approach, already under exploration in diverse fields such as non-alcoholic liver disease, diabetes mellitus, myocardial fibrosis, and resensitization of chemotherapy-resistant cancer cells, holds immense potential (33). However, it is essential to acknowledge the limitations of our current study. Primarily, the outcomes may not be universally applicable, as our sample pool is derived from Xianyou, Fujian. Additionally, future studies necessitate larger sample sizes to ensure a more precise exploration of influencing factors.

Persisting as the primary contributor to cancer-related mortality on a global scale, gastric cancer maintains a dynamic temporal trend. It continues to be characterized by high incidence, elevated morbidity, and a substantial disease burden (34). Our research serves as a foundational reference for future investigations into molecular mechanisms, contributing to a comprehensive understanding of the involvement of single nucleotide polymorphisms (SNPs) in the pathogenesis of gastric cancer.

## Data availability statement

The data analyzed in this study is subject to the following licenses/restrictions: The data are not publicly available due to privacy. Requests to access these datasets should be directed to pingwu_19@163.com.

## Ethics statement

The studies involving humans were approved by the Ethics Committee of Fujian Medical University, China. The studies were conducted in accordance with the local legislation and institutional requirements. The participants provided their written informed consent to participate in this study.

## Author contributions

PW: Writing – original draft. YZ: Validation, Writing – review & editing. YL: Visualization, Methodology, Writing – review & editing. JC: Formal analysis, Conceptualization, Writing – review & editing. YJ: Writing – review & editing, Data curation. JX: Writing – review & editing. BL: Writing – review & editing, Project administration. CW: Writing – review & editing, Supervision, Resources.
